# Pharmacokinetic/Pharmacodynamic Modeling of Spiramycin against *Mycoplasma synoviae* in Chickens

**DOI:** 10.3390/pathogens10101238

**Published:** 2021-09-25

**Authors:** Sara T. Elazab, Nahla S. Elshater, Yousreya H. Hashem, Nayera M. Al-Atfeehy, Eon-Bee Lee, Seung-Chun Park, Walter H. Hsu

**Affiliations:** 1 Department of Pharmacology, Faculty of Veterinary Medicine, Mansoura University, Mansoura 35516, Egypt; sarataha1@mans.edu.eg or; 2 Reference Laboratory for Veterinary Quality Control on Poultry Production, Animal Health Research Institute, Agriculture Research Center, Giza 12618, Egypt; nahlaelshater80@gmail.com (N.S.E.); hanya_noour@yahoo.com (N.M.A.-A.); 3 Mycoplasma Research Department, Animal Health Research Institute, Agriculture Research Center, Giza 12618, Egypt; yousreya@gmail.com; 4 Laboratory of Veterinary Pharmacokinetics and Pharmacodynamics, College of Veterinary Medicine, Kyungpook National University, Daegu 41566, Korea; eonbee@gmail.com (E.-B.L.); parksch@knu.ac.kr (S.-C.P.); 5 Department of Biomedical Sciences, College of Veterinary Medicine, Iowa State University, Ames, IN 50011, USA

**Keywords:** spiramycin, HPLC, pharmacokinetics, withdrawal time, pharmacodynamics

## Abstract

This research aimed to assess the pharmacokinetics/pharmacodynamics (PK/PD) and tissue residues of spiramycin in chickens. The PK of spiramycin were determined in 12 chickens using a parallel study design in which each group of chickens (*n* = 6) received a single dose of spiramycin at 17 mg/kg intravenously (IV) or orally. Plasma samples were collected at assigned times for up to 48 h to measure spiramycin concentrations. Additionally, a tissue depletion study was performed in 42 chickens receiving spiramycin at 17 mg/kg/day orally for 7 days. The area under the plasma concentration–time curve values were 29.94 ± 4.74 and 23.11 ± 1.83 µg*h/mL after IV and oral administrations, respectively. The oral bioavailability was 77.18%. The computed withdrawal periods of spiramycin were 11, 10, and 7 days for liver, muscle, and skin and fat, respectively. The minimum inhibitory concentration for spiramycin against *Mycoplasma synoviae* (*M. synoviae*) strain 1853 was 0.0625 µg/mL. Using the PK/PD integration, the appropriate oral dose of spiramycin against *M. synoviae* was estimated to be 15.6 mg/kg. Thus, we recommend an oral dose of 15.6 mg spiramycin/kg against *M. synoviae* in chickens and a withdrawal period of 11 days following oral treatment with 17 mg spiramycin/kg/day for 7 days.

## 1. Introduction

Spiramycin, a 16-membered ring macrolide antimicrobial drug, was originally isolated from *Streptomyces ambofaciens* in 1954 [1]. It consists of three components: spiramycin I (~63%), spiramycin II (~24%), and spiramycin III (~13%), showing different C-3 substituents [2,3]. Spiramycin displays excellent efficacy in combating several microorganisms, including Gram-positive bacteria, *Chlamydiae*, *Mycoplasma*, *Toxoplasma gondii*, and *Cryptosporidium* species [4,5]. The bacteriostatic effect of spiramycin is attributable to its inhibitory action on protein synthesis via blocking the 50S subunit of the bacterial ribosome [6]. Spiramycin is usually prescribed in veterinary medicine as a therapy for respiratory diseases in birds and ruminants, mastitis, arthritis, and foot-rot in cattle, and infectious gastroenteritis in pigs [7]. In addition, it shows prominent effect against *Mycoplasma* infection in chickens [8].

Similar to other macrolides, spiramycin is recognized by its high tissue distribution [9]. The pharmacokinetics (PK) of spiramycin have been assessed in cattle [7,10], pigs [11], ewes [12], and monkeys [13]. To date, no reports exist concerning the PK of spiramycin in chickens, despite the fact that it is efficacious in the control of mycoplasmosis in this species [8].

*Mycoplasma synoviae* (*M. synoviae*) is a widespread and economically significant pathogen of birds. It causes subclinical respiratory disease and the infection may spread systemically throughout the body if accompanied by a secondary infection to invade the joints and tendons, resulting in synovitis in chickens and turkeys [14,15,16]. Affected birds with infectious synovitis usually exhibit depression, lameness, and swollen hock joints [17]. Horizontal and vertical transmission are the main routes of *M. synoviae* dissemination [18]. *M. synoviae* causes considerable economic losses to the poultry industry as a result of reduced hatchability, drop in egg production, and carcass condemnation [15,19,20]. Spiramycin has been recommended as an effective remedy for mycoplasmosis in chickens [8]. Adjustment and optimization of the spiramycin dosage regimen is essential to maximize its antimicrobial activity and to prevent the development of drug resistance.

Pharmacokinetic/pharmacodynamic (PK/PD) integration provides a powerful tool for establishing rational dosage schedules for antibacterial drugs that achieve the desirable therapeutic outcomes and avoid toxicity and the emergence of resistant bacterial strains [21,22]. It has been reported that high doses of spiramycin may cause gastrointestinal disturbances and allergic reactions [23]. To the best of our knowledge, the PK/PD modeling of spiramycin against *M. synoviae* has not been established.

The indiscriminate usage of antimicrobial agents and/or disregarding the withdrawal period instructions after treatment lead to accumulation of drug residues in edible products obtained from treated animals [24,25]. Such drug residues may be associated with adverse reactions in consumers such as allergies, direct toxicity, imbalance of commensal intestinal microflora, bone marrow depression, various organ diseases, teratogenicity, or cancer [26]. In particular, the existence of macrolide antibiotics residues in the edible tissues has been implicated in the occurrence of hypersensitivity manifestations in sensitive persons, in addition to the alterations of gut microflora [27]. Moreover, the antibiotic residues may enhance the resistance of pathogens [28]. Hence, the World Health Organization identifies the Maximum Residual Limits (MRL) for all antimicrobials in various food products to guarantee consumers’ safety. The Committee for Veterinary Medicinal Products (CVMP) of the European Union (EU) reports the MRL for spiramycin in tissues of chickens to be 0.2 µg/g for muscles, 0.3 µg/g for skin and fat, and 0.4 µg/g for liver [29]. The depletion of spiramycin residues from tissues has been studied in cattle [10], buffalo [30], and fish [31]. However, limited data are available about the depletion of spiramycin from chicken tissues [32].

Thus, this study was undertaken to: (a) investigate the PK characteristics of spiramycin in healthy chickens after oral and intravenous (IV) administrations; (b) investigate the antimicrobial activity of spiramycin against *M. synoviae*; (c) estimate the dose of spiramycin against *M. synoviae* in chickens using the AUC_24h_/MIC (the ratio of the area under the concentration–time curve from 0–24 h: minimum concentration of spiramycin inhibiting the examined strain of *M. synoviae*) for PK/PD integration, and (d) evaluate spiramycin residues in tissues (muscle, skin and fat, and liver) of chickens treated orally with the clinical dose of spiramycin and calculate its withdrawal period in chickens.

## 2. Results

### 2.1. Spiramycin PK

**Figure 1:**
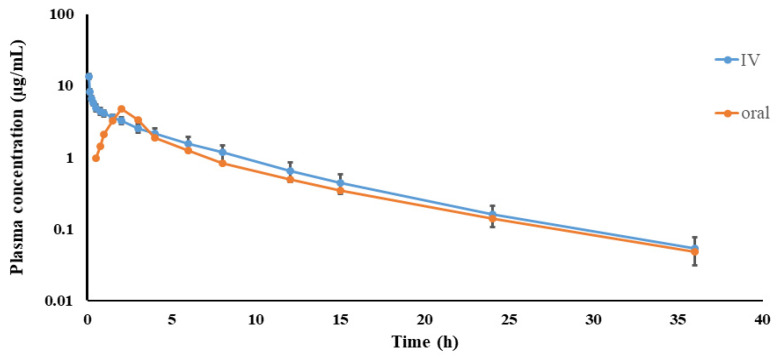
The concentration–time plot for spiramycin in plasma after IV and oral administrations in chickens at 17 mg/kg. Values are presented as mean ± SEM (*n* = 6).

Spiramycin was quantifiable in the plasma for 36 h after IV and oral administrations, respectively, at a single dose of 17 mg/kg. No spiramycin concentration could be detected thereafter. Concentration vs. time profiles of spiramycin in plasma after both routes of administration in chickens are depicted on a semilogarithmic graph in Figure 1. The mean (±SEM) PK parameters of spiramycin are illustrated in Table 1. The values of the area under the plasma concentration–time curve (AUC_0-last_) were 29.94 ± 4.74 and 23.11 ± 1.83 µg*h/mL after IV and oral administrations, respectively. The oral bioavailability (F) was 77.18%. No statistical difference was noticed in the PK parameters between IV and oral routes.

### 2.2. Plasma Protein Binding

The mean value of plasma protein binding ratio of spiramycin was 34.60 ± 1.28%. 

### 2.3. Spiramycin PD

The minimum inhibitory concentration (MIC) of spiramycin against *M. synoviae* strain (MS WVU-1853) was 0.0625 µg/mL. 

**Figure 2:**
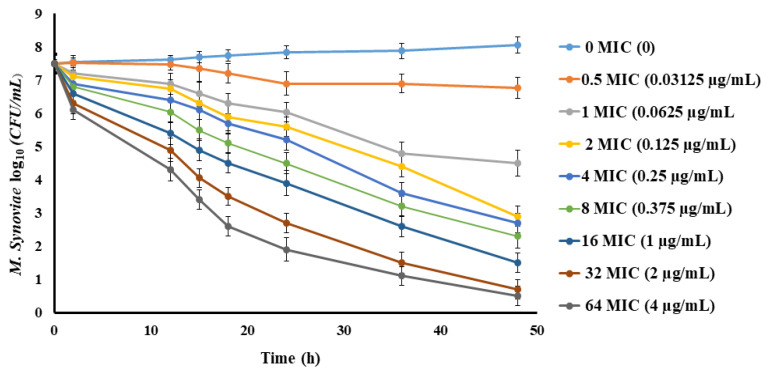
In vitro time-kill plots for spiramycin concentrations ranging 0–64 MIC against *M. synoviae* in the growth medium. Values are shown as mean ± SEM (*n* = 3).

The in vitro anti-mycoplasmal activities of multiples of MIC (0.5–64 MIC) of spiramycin against *M. synoviae* through a period of 48 h is shown in Figure 2. On the whole, a time-dependent feature of spiramycin against *M. synoviae* was noticed from the killing profiles as the rate of killing increased by increasing the time that *M. synoviae* was subjected to spiramycin application. In addition, the killing curves revealed a concentration-dependent effect of spiramycin against *M. synoviae* as the anti-mycoplasmal effect increased in response to the rise in the spiramycin concentration (1–64 MIC), where 64 MIC displayed the maximal activity (7.56 log_10_CFU/mL) decline during the 48 h incubation.

**Figure 3:**
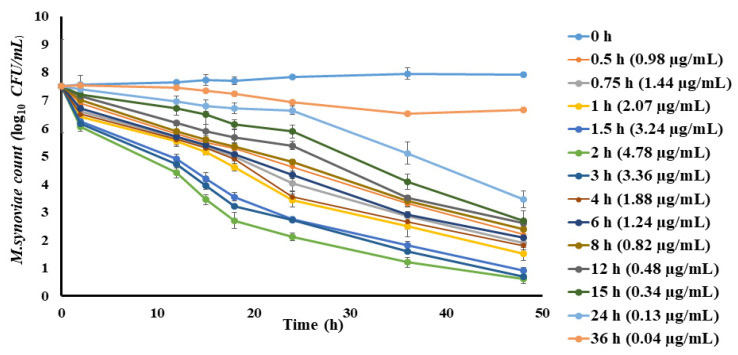
Ex vivo inhibition of *M. synoviae* growth in plasma after oral administration of spiramycin (sampling times of 0, 0.5, 0.75, 1, 1.5, 2, 3, 4, 6, 8, 12, 15, 24, 36 h). Values are exhibited as mean ± SEM (*n* = 6). Values (µg/mL) in brackets are the corresponding spiramycin concentration in the samples at various time points shown as mean ± SEM.

The ex vivo anti-mycoplasmal effect of spiramycin against *M. synoviae* was assessed utilizing plasma samples collected from chickens before and at 0.5, 0.75, 1, 1.5, 2, 3, 4, 6, 8, 12, 15, 24, and 36 h after oral administration of spiramycin (when mean spiramycin concentrations were 0, 0.98, 1.44, 2.07, 3.24, 4.78, 3.36, 1.88, 1.24, 0.82, 0.48, 0.34, 0.13, and 0.04 µg/mL, respectively). Figure 3 shows the ex vivo time-killing curves. For plasma samples obtained from chickens between 0.5 and 8 h, spiramycin produced a ≥3 log_10_ CFU/mL reduction in the *M. synoviae* count after 24 h of exposure. Meanwhile, for samples collected at 12, 15, 24, and 36 h, spiramycin application resulted in a ≤2 log_10_ CFU/mL decline in the count following 24 h of incubation. 

### 2.4. PK/PD Integration and Dose Estimation

**Figure 4:**
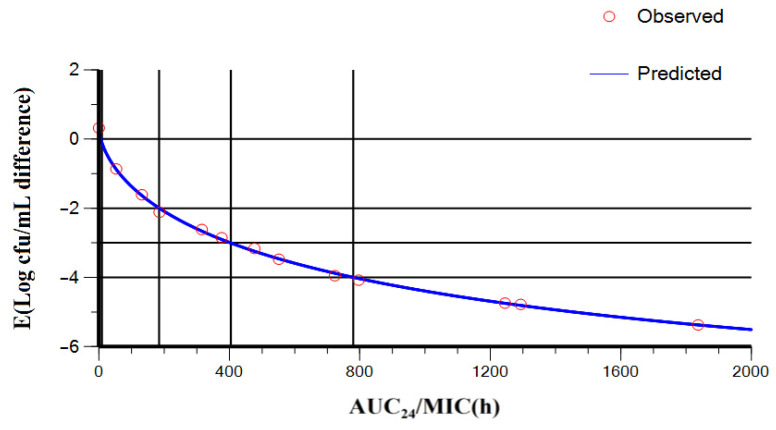
Sigmoidal E_max_ correlation for the *Mycoplasma* count (Log_10_CFU/mL) vs. AUC_24h_/MIC Figure 2. Integration of pharmacokinetic and pharmacodynamic data obtained for spiramycin after administration at 17 mg/kg in chickens.

The correlation between the PK/PD parameters derived from dynamic model assays and anti-mycoplasmal activity are presented in Figure 4. The parameters obtained from the PK/PD modeling of ex vivo spiramycin data for plasma are illustrated in Table 2.

The estimated AUC_24h_/MIC needed to obtain mycoplasmastasis (E = 0, no difference in bacterial count after incubation for 24 h), mycoplasmocide (E = −2 and E = −3, 99 and 99.9% reduction in the *M. synoviae* count, respectively) and the virtual mycoplasmal eradication (E = −4) were 10, 185, 405, and 780 h, respectively. Hence, the dose of spiramycin required daily for the bacteriostatic action (E = 0), bactericidal action (E = −2 and E = −3) and bacterial elimination (E = −4) against *M. synoviae* in chickens were calculated to be 0.84, 15.60, 34.18, and 65.83 mg/kg, respectively.

### 2.5. Tissue Residues and Withdrawal Periods of Spiramycin

**Figure 5:**
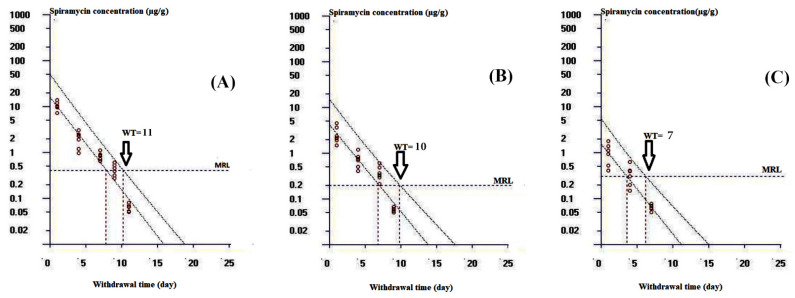
The withdrawal time (WT) determination at the time when the one-sided 95% upper tolerance limit was below the EU MRL of 0.2 µg/g for muscle, 0.3 µg/g for skin and fat, and 0.4 µg/g for liver. (**A**) exhibits the withdrawal time estimation for chicken liver; (**B**) displays the withdrawal time evaluation for chicken muscle; (**C**) shows the withdrawal time calculation for chicken skin and fat.

Results from tissue residue analysis of chickens receiving spiramycin orally at 17 mg/kg for 7 consecutive days revealed that liver samples had the highest spiramycin concentration, followed by the muscle, while the lowest levels were found in skin and fat samples. The residue concentrations of spiramycin were above the limit of quantification (LOQ) up to the eleventh, ninth, and seventh days after the last dose in liver, muscle, and skin and fat, respectively. The mean spiramycin residue levels in chicken tissues are listed in Table 3. The computed withdrawal periods were 11, 10, and 7 days for liver, muscle, and skin and fat, respectively, as displayed in Figure 5A–C.

## 3. Discussion

Despite extensive marketing of spiramycin in poultry medicine, PK data in birds are absent. To the best of our knowledge, this is the first report about PK/PD modeling of spiramycin against *M. synoviae* in chickens. The PK analysis in the present study demonstrated that following IV injection of spiramycin in chickens at 17 mg/kg, the apparent volume of distribution (Vz_obs) was 3.3 L/kg which illustrated its wide distribution in the intracellular and extracellular compartments of the body. This finding coincides with the view of Riviere [33] who indicated that a Vz higher than 1 L/Kg represents a wide diffusion of the drug throughout the tissues. The high lipid solubility and the moderate plasma protein binding features of spiramycin are probably the main reasons beyond its high Vz. However, the value found for Vz in this study was lower than that reported in pigs (5.6 L/Kg) receiving IV spiramycin at 10 mg/kg [11], ewes (16.56 L/kg) receiving IV spiramycin at 20 mg/kg [12], calves (23.5 L/kg) receiving spiramycin IV at 15 mg/kg [34], and cattle (11.97 L/Kg) receiving spiramycin IV at 15.6 mg/kg [35].

In addition, the clearance (Cl_obs) value of spiramycin obtained from this research (0.68 L/h/kg) was in line with that found in ewes (0.757 L/h/kg) [12]. However, this value was lower than that published for cows (1.18 L/h/kg) [7], and pigs (2.6 L/h/kg) [11] receiving IV spiramycin at 10 mg/kg. These variations may be due to the species difference. 

The findings of the present study displayed a short elimination half-life (T_1/2λz_^)^ (3.97 h) for spiramycin after IV injection, which reflected its rapid distribution into tissues or elimination in chickens. This T_1/2λz_ was shorter than that reported in cows (8.61 h) [7]. This disparity may be explained by the species difference in the metabolic rate. Poultry are known to have a higher metabolic rate than mammals, which may account for the rapid metabolism of spiramycin in birds [36]. The AUC extrapolated to infinity was 30.25 µg*h/mL after IV administration. A similar value was observed in ewes (28.22 µg*h/mL) [11].

Following oral administration, spiramycin was rapidly absorbed from the gut with a peak plasma concentration (C_max_) of 4.78 µg/mL achieved at 2 h (the time to accomplish C_max_ (T_max_)). These results are consistent with Nielsen and Gyrd-Hansen [11], who reported a C_max_ of 5.2 µg/mL attained at 2 h after oral administration of spiramycin at 55 mg/kg in pigs. Moreover, in pigs receiving spiramycin in feed at 85 mg/kg. a C_max_ of 4 µg/mL was reported [37]. On the basis of the calculated AUC_0-last_ from the present study, the oral bioavailability of spiramycin in chickens was calculated as 77.18%. This bioavailability is comparable to that reported for pigs (60%) [11]. Furthermore, the mean residence time (MRT) (6.63 h) was similar to that observed in pigs (7.2 h) [11].

Previous reports have elucidated the importance of plasma protein binding in PK as it plays a pivotal role in the distribution and the excretion of drugs [38,39]. Since the ratio of plasma protein binding differs between animal species, it is crucial to consider protein binding in the target species in the adjustment of volume and clearance [40,41]. A considerable variation was noticed in plasma protein binding among members of the macrolide antibiotic family [42]. Herein, spiramycin was 34.6% bound to plasma proteins in chickens. This ratio was comparable to that previously recorded for tylosin (32.40%), a macrolide antibiotic, in duck plasma [43]. However, this percentage was higher than that reported for tylvalosin (13%) in turkey plasma [44]. To the best of our knowledge, this is the first report regarding the plasma protein biding of spiramycin.

Although macrolides have been recognized as time-dependent drugs [45,46], recent studies have proved a concentration dependency for some members of this class. Tulathromycin expressed a concentration-dependent action against *Pasteurella multocida* [47]. Moreover, Huang et al. [48] reported a concentration-dependent activity for tilmicosin against *Mycoplasma gallisepticum* in vitro. Moreover, a few macrolides (e.g., azithromycin) exhibit both a time- and concentration-dependent killing effect [49]. Prior investigation revealed that the kind of bacterial killing effect (either time- or concentration-dependent activity) of antibiotics may vary under different circumstances [50]. In the present study, the dynamics of the bactericidal effect exerted by spiramycin were both time- and concentration-dependent. These observations are in agreement with our previous study [43] in which tylosin, a macrolide antibiotic, displayed a time- and concentration-dependent activity against *Mycoplasma anatis* in vitro. Furthermore, Renard et al. [51] demonstrated a time-dependent activity for spiramycin against *Staphylococcus aureus*.

The time above the MIC (%T > MIC) index is markedly correlated with the antimicrobial efficacy of macrolides [52,53]. Nevertheless, for azithromycin, the AUC_24h_/MIC ratio is the favorable PK/PD parameter utilized to elucidate its antibacterial activity [54]. In the present study, the PK/PD findings demonstrated that the AUC_24h_/MIC had the best correlation with the anti-mycoplasmal activity (correlation coefficient (R^2^) = 0.9628). In accordance with that of Emam et al. [55], the MIC of spiramycin against *M. synoviae* strain (MS WVU-1853) was estimated to be 0.0625 µg/mL. On the basis of this MIC, the obtained AUC_24h_/MIC for mycoplasmacidal activity (185 h), the evaluated unbound portion of spiramycin in chicken plasma (65.4%), and the oral bioavailability (77.1%), the appropriate daily dosage of spiramycin needed to achieve a mycoplasmacidal effect (2 log_10_ decline in the *Mycoplasma* count) against *M. synoviae* in chickens was calculated to be 15.6 mg/kg/day. This calculated dose is comparable to the one recommended by the manufacturer (17 mg/kg/day) for the treatment of mycoplasmosis in poultry.

As presented in Figure 4, the administered spiramycin dose (17 mg/kg) could achieve mycoplasmastasis, mycoplasmacide, and mycoplasmal elimination. These results are consistent with those of Renaudin et al. [56] who reported a bactericidal activity of spiramycin against *Mycoplasma pneumoniae* in vitro. In addition, tilmicosin produced a mycoplasmacidal effect against *M. gallisepticum* in vitro [48].

In this research, the tissue depletion study for spiramycin after oral administration at 17 mg/kg/day for 7 successive days showed that spiramycin was highly concentrated in the liver, followed by the muscle, whereas the fat and skin tissue had the lowest level. Spiramycin was quantifiable in the liver, muscle, and skin and fat tissue up to eleventh, ninth, and seventh day post-medication, respectively. The persistence of spiramycin residues in these tissues for a long time may be attributable to its high tissue penetration property [11]. Our findings are in concordance with the data exhibited in the CVMP report [57], which revealed that after providing spiramycin at 0.8/L in drinking water for chickens for 3 days, spiramycin became undetectable in muscle and skin and fat; spiramycin was undetectable in liver at the 10th day post-last dose.

Considering the computed withdrawal period of spiramycin in the present study for the liver, muscle, and skin and fat tissue, a preslaughter clearance time of 11 days is recommended after oral administration of spiramycin at 17 mg/kg for 7 days in chickens to ensure consumers' safety. Along the same lines, Alsafi and Kafi [32] recommended that chickens not be slaughtered until 8 days after the end of the treatment with spiramycin at 15 mg/kg orally for 5 consecutive days.

In conclusion, our present findings suggested a dose of 15.6 mg/kg/day for spiramycin to be used clinically against *M. synoviae* in chickens. Additionally, a note should be added on the package of spiramycin indicating that an 11-day withdrawal period is required after its administration orally to ascertain the secure consumption of chicken edible tissues. However, this recommended dose is dependent on the findings of the MIC of one strain of *M. synoviae*; it would be preferable to incorporate many diverse strains of *M. synoviae* to take into account the variation in the sensitivity to spiramycin. Moreover, further studies are warranted to assess the PK and the activity of spiramycin in *M. synoviae*-infected chickens as the PK characteristics of antimicrobial agents may vary between the healthy and infected chickens. 

## 4. Materials and Methods

### 4.1. Materials

The current research was executed using spiramycin injectable solution (Mayco Spira^®^, 540,000 IU/mL corresponding to 168.75 mg/mL spiramycin as spiramycin adipate, Laboratorios Maymó, S.A., Barcelona, Spain); it was diluted with sterile 0.9% NaCl to make 56 mg/mL spiramycin for the animal experiment. The reference standard of spiramycin was procured from Sigma-Aldrich Co. (St. Louis, MO, USA). Phosphoric acid, methanol, n-hexane, acetonitrile, and potassium monobasic phosphate were manufactured by Thermo Fisher Scientific (Waltham, MA, USA). Ammonium acetate and chloroform were bought from Merck (Darmstadt, Germany). All utilized chemicals were of high-performance liquid chromatography (HPLC) analytical grade. Purification of water for HPLC was performed employing a Milli-Q system (Waters Corp., Milford, MA, USA). *M. synoviae* reference strain (MS WVU-1853) was provided by Animal Health Research Institute, Giza, Egypt. *M. synoviae* medium base was produced following the method of Frey et al. [58].

### 4.2. Experimental Animals

This investigation was performed using 54 healthy Hubbard broiler chickens of both sexes, nearly 4 weeks old and weighing 1–1.5 kg. Chickens were purchased from the Faculty of Agriculture, Mansoura University, Egypt. They were housed individually in cages under hygienic conditions at 25–27 °C and 60 ± 10% relative humidity with a 12-h light/dark cycle.

The birds were offered a medication-free diet with ad libitum access to water. They underwent a 2-week acclimation period preceding the study. The Research Ethics Committee of the Faculty of Veterinary Medicine, Mansoura University, Mansoura, Egypt, approved all animal procedures (Approval No. R/88).

### 4.3. Experimental Design

For the PK study, 12 chickens were assigned randomly to 2 groups, with 6 chickens in the oral group and 6 chickens in the IV group: 3 females and 3 males. Spiramycin was administered at a single dose of 17 mg/kg [32, 35, and as recommended by the manufacturer] directly into the crop by gavage and into the right wing vein of chickens in the oral and IV groups, respectively. Blood (1 mL) was collected from the left wing vein into heparinized tubes before spiramycin dosing (0 h) and at 0.5, 0.75, 1, 1.5, 2, 3, 4, 6, 8, 12, 15, 24, 36, 48 h after oral administration and at 0.083, 0.167, 0.25, 0.33, 0.5, 0.75, 1, 1.5, 2, 3, 4, 6, 8, 12, 15, 24, 36, 48 h following IV injection. Blood samples were centrifuged at 1257× *g* for 10 min. The produced plasma was promptly stored at −20 °C for later analysis.

For tissue residue study; 42 chickens were given spiramycin orally by gavage at 17 mg/kg once daily for 7 consecutive days [59]. After the 7th day of dosing, 6 (3 females and 3 males) chickens were slaughtered on 1st, 4th, 7th, 9th, 11th, 13th, and 15th days, respectively, post-spiramycin administration. Tissue samples (breast muscle, abdominal skin with fat, and liver) were collected from each chicken and were kept at −20 °C until analysis.

### 4.4. Measurement of Spiramycin Concentrations in Plasma and Tissue Samples

#### 4.4.1. Extraction of Plasma Samples

The extraction procedures of spiramycin from plasma samples were performed as previously described [11] with minor modification. In brief, a 0.5 mL plasma sample was mixed with 250 µL of 0.2 M potassium phosphate buffer (pH 5.8) and 1.5 mL chloroform, and was vortexed for 1 min. Thereafter, centrifugation of the mixture was performed for 20 min at 2600× *g*. Then, the upper aqueous phase was discarded. After evaporation of the chloroform layer to dryness at 40 °C under air stream, the residue was redissolved in the mobile phase (0.05 M phosphoric acid-acetonitrile (75: 25, pH 3.0, *v*/*v*)). After the extraction of the analyte, 100 µL of the sample was injected into the HPLC device.

#### 4.4.2. Extraction of Tissue Samples

Spiramycin was extracted from tissue samples as previously described [31]. After extraction, 100 µL of the sample was injected into the HPLC apparatus.

#### 4.4.3. Chromatographic Conditions

The analysis of spiramycin in plasma and tissue samples was conducted by a method adapted from Lee et al. [31] with minor modifications. Spiramycin concentrations were measured using HPLC Agilent Series 1200 quaternary pump, Series 1200 autosampler, Series 1200 UV VIS detector set at 232 nm, and HPLC 2D ChemStation software (Hewlett-Packard, Les Ulis, France). Separation was achieved using a Phenomenex C18 column (5 µm, 250 × 4.6 mm) at 35 °C and 0.05 M phosphoric acid-acetonitrile in the ratio of 75: 25 (pH 3.0, *v*/*v*) as the mobile phase at a flow rate of 1.5 mL/min. The retention time was 3.17 min. The HPLC technique was validated (by determining recovery, precision, sensitivity, and linearity) as stated in the European Medicines Agency report [60] (Table 4). The lower limits of detection and quantification of spiramycin were 0.003 and 0.01 µg/mL in plasma, and 0.02 and 0.05 µg/g in tissue samples, respectively.

### 4.5. Plasma Protein Binding Determination

In vitro protein binding assay was performed utilizing plasma samples obtained from chickens before spiramycin administration. The ultrafiltration method was employed as previously reported [61]. Five replicates of 1 mL plasma samples were spiked with spiramycin at concentrations of 0.05, 0.1, 0.5, 1, and 2.5 µg/mL. One mL of each sample was placed in a Pierce protein concentrator PES, 30 MWCO (Thermo Fisher Scientific, USA). Centrifugation of samples was carried out at 2000× *g* for 10 min. Plasma samples and ultrafiltrates were injected into HPLC column for analysis. The ratio of plasma protein binding was determined using the following equation:

(1) $$\mathrm{Protein}\;\mathrm{binding}\;(\%)=\frac{\left(\mathrm{Total}\;\mathrm{concentration}-\mathrm{Ultrafiltrate}\;\mathrm{concentration}\right)\times 100}{\mathrm{Total}\;\mathrm{concentration}}$$

### 4.6. Determination of MIC of Spiramycin against M. synoviae

The MIC of spiramycin against *M. synoviae* strain (MS WVU-1853) was determined utilizing a previously described method [48,62]. In brief, cultures of *M. synoviae* were diluted with the mycoplasma medium to 10^7^ CFU/mL. A series of spiramycin concentrations were prepared in 96-well plate (final concentration, 0.03125 to 32 µg/mL). *M. synoviae* at 10^7^ CFU/mL was added into each well in a similar amount of medium (100µL). In addition, a sterility control (sterile *M. synoviae* broth at pH 7.8), a growth control (*M. synoviae* inoculum without antimicrobial), and an end point control (blank medium at pH 6.8) were also utilized. The humidified incubator was set at 37 °C, and 5% CO_2_ for the cultured plates. The MIC was evaluated as the lowest concentration of spiramycin that caused no change in the color of culture medium. All the experiments were conducted in triplicate.

### 4.7. In Vitro Time-Killing Curve 

The in vitro time-killing investigations were carried out as reported [63]. Briefly, spiramycin was used at 0, 0.5, 1, 2, 4, 8, 16, 32, and 64 MIC. In a penicillin bottle, the culture system consisted of 3.5 mL blank medium, 0.1 mL of spiramycin solution, and 0.4 mL suspension of *M. synoviae* in the exponential phase. The final inoculum was 10^7^ CFU/mL. The penicillin bottles were kept in the incubator for 48 h with 5% CO_2_, and at 37 °C. Samples (100 μL) were obtained from each bottle at 0, 2, 12, 15, 18, 24, 36, and 48 h, to quantify the viable counts of *M. synoviae.* Each spiramycin concentration was examined thrice. For establishing the in vitro time-killing plot, the mean values of log_10_CFU/mL (*n* = 3) were drawn against time (h) with various spiramycin concentrations.

### 4.8. Ex Vivo Time-Killing Curve Determination

The determination of the ex vivo anti-mycoplasmal activity of spiramycin was conducted utilizing plasma samples collected from healthy chickens at 0, 0.5, 0.75, 1, 1.5, 2, 3, 4, 6, 8, 12, 15, 24, 36 h post-oral spiramycin administration. The viable counts of *M. synoviae* were determined using the above-mentioned technique for the in vitro time-killing experiments. The ex vivo time-killing curve illustrated the relationship between the mean values of log_10_CFU/mL (*n* = 6) in plasma samples collected at various time points from chickens receiving spiramycin and the time of incubation (h). 

### 4.9. Assessment of PK Properties and Withdrawal Time Determination

Plasma spiramycin concentrations vs. time plots were investigated for each chicken using a non-compartmental approach [11]. The PK analysis was conducted utilizing WinNonlin 8.3 software (Certara, USA). Following oral administration of spiramycin, the C_max_ and the T_max_¬ were determined. The T_1/2λz_ was calculated via the equation T_1/2λz_ = 0.693/λz, where λz, the first order rate constant, was defined from the slope of the terminal phase of the concentration vs. time curve. The linear trepazoidal rule and the linear-up log-down method were used to calculate AUC_0-∞_ after IV and oral administrations of spiramycin, respectively. The Cl-obs was determined as Cl-obs = dose/AUC. In addition, the Vz-obs was calculated as Vz = dose/(λz × AUC). The F was obtained by dividing mean AUC_oral_ by mean AUC_IV_.

The concentrations of spiramycin in the collected tissues were utilized to determine preliminary withdrawal times using linear regression approach by applying WT 1.4 software, designed in Germany and licensed by the CVMP of EU as previously reported [64]. The withdrawal time (WT) was defined to be the time when the upper one-sided tolerance limit (95%) with 95% confidence interval was below the MRL of spiramycin which was determined by CVMP in chicken tissues to be 0.2 µg/g for muscle, 0.3 µg/g for skin and fat, and 0.4 µg/g for liver [29].

### 4.10. PK/PD Integration and Dose Computation

For PK/PD modeling, the AUC_24h_/MIC values were computed based on the area under the plasma concentration curve over 24 h divided by the MIC. The AUC_24h_ values were determined by multiplying the plasma spiramycin concentration at each time point of collection after oral administration by the incubation period (24 h) [47,65].

The sigmoidal inhibitory E_max_ model [WinNonlin 8.3 software (Certara, USA)] was applied to correlate the ex vivo AUC_24h_/MIC with the log_10_ difference between the initial *M. synoviae* count and that after 24 h of incubation. This model was elucidated by the following equation:

E = E_0_ − [(E_max_ × C ^γ^)/(C^γ^ + EC^γ^_50_)] (2)

where E denotes the anti-mycoplasmal activity measured as the change in the *M. synoviae* count (log_10_CFU/mL) in the plasma sample after 24 h, compared to the baseline log_10_CFU/mL (the growth without spiramycin; E_max_ points out the maximum anti-mycoplasmal effect; E_0_, reflects the variation in *M. synoviae* count in the control samples (without spiramycin) between 0 and 24 h. EC_50_ indicates AUC/MIC ratio producing 50% reduction in *M. synoviae* counts from the initial count; C represents AUC/MIC in the effect compartment, and γ is the Hill coefficient which accounts for the steepness of the AUC_24h_/MIC effect curve. 

Three levels of anti-mycoplasmal effect of spiramycin were assessed by estimating the values of AUC_24h_/MIC required for bacteriostatic action (E = 0), bactericidal action (E = −2 and E = −3), and for bacterial elimination (E = −4).

The appropriate daily dose of spiramycin in chickens was calculated on the basis of the following equation:

(3) $$\mathrm{Dose}\;\left(perday\right)=\frac{\left({\mathrm{AUC}}_{24}/\mathrm{MIC}\right)breakpoint\times MIC\times Clearance\;\left(per\;h\right)}{fu\times F}$$

In which AUC_24h_/MIC elucidates the ex vivo AUC_24h_/MIC value for optimal activity; MIC indicates the minimum inhibitory concentration; CL reflects the clearance; F is the bioavailability, and fu refers to the free part of spiramycin in plasma

### 4.11. Statistical Analysis

Data are presented as mean ± SEM. A Shapiro–Wilk test was used to check the normality of the data. The main PK parameters after oral and IV administrations were compared using Wilcoxon’s rank sum test. Furthermore, the concentrations of spiramycin were statistically analyzed in different tissues utilizing ANOVA. Mean comparisons were performed using Tukey’s test. Differences were considered significant when *p* < 0.05. Statistical comparison was carried out by Statistical Package for Social Science (SPSS), version 20 (SPSS Inc., Chicago, IL, USA).

## Figures and Tables

**Table 1:** Pharmacokinetic parameters of spiramycin in chickens after IV and oral administrations at 17 mg/kg.

Parameters	IV	Oral
C_max_ (µg/mL)	NA	4.78 ± 0.30
T_max_ (h)	NA	2.00 ± 0.00
λz (1/h)	0.22 ± 0.04	0.12 ± 0.02
T_1/2 λz_ (h)	3.97 ± 0.85	6.25 ± 0.90
AUC_0-last_ (µg*h/mL)	29.94 ± 4.74	23.11 ± 1.88
AUC_0-∞_ (µg*h/mL)	30.25 ± 4.78	23.61 ± 1.90
Vz_obs (L/kg)	3.30 ± 0.52	--------
Vz_F_obs (L/kg)	--------	6.58 ± 0.89
Cl_obs (L/h/kg)	0.68 ± 0.15	--------
Cl_F_obs (L/h/kg)	--------	0.74 ± 0.05
MRT (h)	5.04 ± 0.91	6.63 ± 0.43
F (%)	--------	77.18

C_max_: The highest plasma concentration; T_max_: time to peak concentration; λz: the first order rate constant; T_1/2λz_: elimination half-life; AUC_0-last_: area under the plasma concentration vs. time curve from 0 to last time; AUC_0-∞_: area under the plasma concentration–time plot from 0 to infinite; Vz_obs: apparent volume of distribution in terminal phase; Vz_F_obs: volume of distribution scaled by bioavailability; Cl_obs: total body clearance; Cl_F_obs: clearance divided by bioavailability; MRT: mean residence time; F: mean systemic bioavailability; data are expressed as mean ± SEM (*n* = 6); NA = not applicable.

**Table 2:** Integration of pharmacokinetic and pharmacodynamic data obtained for spiramycin after administration at 17 mg/kg in chickens.

Parameter	Units	Mean ± SEM
E_max_	CFU/mL	10.13 ± 0.97
EC_50_	h	1241.46 ± 355.73
E_0_	CFU/mL	0.32 ± 0.06
E_max_-E_0_	h	9.81 ± 0.91
AUC _24 h_/MIC for bacteriostatic effect (E = 0)	h	10
AUC _24 h_/MIC for bactericidal effect (E = −2)	h	185
AUC _24 h_/MIC for bactericidal effect (E = −3)	h	405
AUC _24 h_/MIC for bacterial elimination (E = −4)	h	780

E_max_, is the maximum antibacterial effect identified as difference in log_10_ CFU/mL in samples over 24 h of incubation with spiramycin; EC_50_, is the PK/PD parameter of the drug that induced 50% of the maximal antibacterial effect; E_0_, is the corresponding mycoplasmal growth in the absence of spiramycin (control samples); AUC_24h_/MIC, values needed to produce bacteriostatic effect and bactericidal effect.

**Table 3:** Concentrations (µg/g) of spiramycin in chicken tissues (liver, muscle, skin and fat) following its oral administration at 17 mg/kg for 7 consecutive days.

Time Post Last Dose (Days)	Liver	Muscle	Skin and Fat
1	10.45 ± 0.88 ^a^	2.66 ± 0.47 ^b^	1.03 ± 0.21 ^b^
4	1.99 ± 0.32 ^a^	0.73 ± 0.11 ^b^	0.34 ± 0.07 ^b^
7	0.85 ± 0.06 ^a^	0.38 ± 0.06 ^b^	0.06± 0.01 ^c^
9	0.43 ± 0.05 ^a^	0.05 ± 0.02 ^b^	ND
11	0.07 ± 0.02	ND	ND
13	ND	ND	ND

Data are shown as mean ± SEM (*n* = 6). Different superscripts within each row indicate significant differences (*p* < 0.05). ND = not detectable (<0.05 µg/g).

**Table 4:** Validation parameters of the HPLC assay.

Analyte	Average Recovery (%)	Precision		Linearity and Range			Sensitivity	
		Intra-Day RSD (%)	Inter-Day RSD (%)	Linearity Concentration Range	Calibration Equation	Correlation Coefficient (R^2^)	LOD	LOQ
Plasma	101.93 ± 2.15	2.6	2.7	0.01–20 µg/mL	y = 142.73x + 0.2289	0.9999	0.003	0.01 µg/mL
Muscle	91.69 ± 4.13	2.9	3.8	0.05–20 µg/g	y = 99.938x − 2.3052	0.9954	0.02	0.05 µg/g
Skin and fat	95.20 ± 3.56	3.3	3.4	0.05–20 µg/g	y = 103.88x − 2.7223	0.9994	0.02	0.05 µg/g
Liver	92.09 ± 4.40	3.4	3.9	0.05–20 µg/g	y = 98.526x − 2.6683	0.995	0.02	0.05 µg/g

Data for recovery are presented as mean ± SEM. Average recovery % for plasma (using spiked concentrations in the range of 0.01–20 μg/mL in triplicate analysis). Average recovery % for muscle, skin, fat, and liver tissues (using spiked concentrations in the range of 0.05–20 μg/g in triplicate analysis. Intra-day relative standard deviation (RSD) and inter-day RSD % for plasma (0.01 µg/mL, *n* = 6). Intra-day RSD and Inter-day RSD % for muscle, skin and fat, and liver tissues (0.05 µg/g, *n* = 6).

## Data Availability

The data sets generated for this study are available from the corresponding author upon reasonable request.
